# Dexmedetomidine Promotes Lipopolysaccharide-Induced Differentiation of Cardiac Fibroblasts and Collagen I/III Synthesis through α_2A_ Adrenoreceptor-Mediated Activation of the PKC-p38-Smad2/3 Signaling Pathway in Mice

**DOI:** 10.3390/ijms222312749

**Published:** 2021-11-25

**Authors:** Jia Liao, Kaiying Li, Xingyu Su, Yihua Chen, Yingwei Wang, Xiangxu Tang, Yun Xing, Yaqian Xu, Xiaomeng Dai, Jiashuo Teng, Hongmei Li, Huadong Wang, Xiuxiu Lv, Yiyang Wang

**Affiliations:** Department of Pathophysiology, School of Medicine, Jinan University, Guangzhou 510632, China; liaojia@stu2019.jnu.edu.cn (J.L.); careyli@stu2016.jnu.edu.cn (K.L.); sxy1998@stu2020.jnu.edu.cn (X.S.); yatwachan037@stu2018.jnu.edu.cn (Y.C.); wangyingwei@stu2019.jnu.edu.cn (Y.W.); xiangxutang@stu2017.jnu.edu.cn (X.T.); xingyun@stu2018.jnu.edu.cn (Y.X.); xuyaqian@stu2018.jnu.edu.cn (Y.X.); 1625041006@stu2016.jnu.edu.cn (X.D.); tengjs826@stu2018.jnu.edu.cn (J.T.); lihongmei@jnu.edu.cn (H.L.); owanghd@jnu.edu.cn (H.W.)

**Keywords:** dexmedetomidine, cardiac fibroblast, differentiation, lipopolysaccharide, α_2_ adrenergic receptor

## Abstract

Dexmedetomidine (DEX), a selective α_2_ adrenergic receptor (AR) agonist, is commonly used as a sedative drug during critical illness. In the present study, we explored a novel accelerative effect of DEX on cardiac fibroblast (CF) differentiation mediated by LPS and clarified its potential mechanism. LPS apparently increased the expression of α-SMA and collagen I/III and the phosphorylation of p38 and Smad-3 in the CFs of mice. These effects were significantly enhanced by DEX through increasing α_2A_-AR expression in CFs after LPS stimulation. The CFs from α_2A_-AR knockout mice were markedly less sensitive to DEX treatment than those of wild-type mice. Inhibition of protein kinase C (PKC) abolished the enhanced effects of DEX on LPS-induced differentiation of CFs. We also found that the α-SMA level in the second-passage CFs was much higher than that in the nonpassage and first-passage CFs. However, after LPS stimulation, the TNF-α released from the nonpassage CFs was much higher than that in the first- and second-passage CFs. DEX had no effect on LPS-induced release of TNF-α and IL-6 from CFs. Further investigation indicated that DEX promoted cardiac fibrosis and collagen I/III synthesis in mice exposed to LPS for four weeks. Our results demonstrated that DEX effectively accelerated LPS-induced differentiation of CFs to myofibroblasts through the PKC-p38-Smad2/3 signaling pathway by activating α_2A_-AR.

## 1. Introduction

Myocardial fibrosis is characterized by net accumulation of extracellular matrix proteins in the cardiac interstitium and results in both systolic and diastolic dysfunctions [1]. It is a common feature of advanced coronary heart disease, hypertension and cardiomyopathy, which results in an increased risk of morbidity and mortality [2,3]. Lipopolysaccharide (LPS), an important component of gram-negative bacteria, also induces cardiac fibrosis, which commonly occurs in sepsis [4,5]. LPS induces the activation of cardiac fibroblast (CF) through Toll-like receptor, leading to the transcription and release of TNF-α, which has been reported to contribute to the progression of septic cardiac dysfunction [6,7].

Dexmedetomidine (DEX), a highly selective α_2_-AR agonist, has gained more popularity in the intensive care management of patients due to its sedative, analgesic, and anxiolytic properties [8]. In 1999, the United States Food and Drug Administration approved DEX as a short-term sedative and analgesic agent for patients on mechanical ventilation in the intensive care unit (ICU) [9]. However, clinical trials have demonstrated that 90-day mortality is increased in septic patients with DEX treatment [10]. Shehabi et al. demonstrated that DEX-based sedation provides some undesirable cardiovascular effects, such as bradycardia and hypotension [11]. It has also been demonstrated that α_2_-AR antagonists have an inhibitory effect on the progression of hepatic fibrosis [12]. The downregulation of α_2_-AR expression in kidneys effectively offsets interstitial fibrosis [13]. However, the potential effect of DEX on LPS-induced cardiac fibrosis has not been well investigated.

Cardiac fibroblasts, which account for 60–70% of the cells in the heart, are a key source of the extracellular matrix (ECM) that regulates the structure of the heart and hence mechanical, chemical and electrical signals between the cellular and non-cellular components [14,15]. The major structural protein within the ECM is fibrillar collagen, which surrounds bundles of myocytes to generate a stress-tolerant network. The myocardial collagen network mainly consists of types I and III collagen, whose proportions are 85% and 11%, respectively [16,17,18]. CFs are critical not only to normal myocardial function but also play an important role in the development of cardiomyopathy. During heart injury, injurious factors lead to CF differentiation into cardiac myofibroblasts, which express alpha-smooth muscle actin (α-SMA) and contribute to the development of cardiac fibrosis. Cardiac myofibroblasts synthesize more collagen and form scar tissue that replaces the damaged lesions, resulting in myocardial fibrosis [3,19]. Myocardial fibrosis protects cardiac function by reducing cardiac stress and maintaining the electromechanical integrity of the heart. However, cardiac compliance and diastolic function decline with the aggravation of myocardial fibrosis, which finally leads to cardiac insufficiency and even heart failure [20,21]. Although there is no α_2_-AR on cardiomyocytes [22], norepinephrine significantly induces protein synthesis and proliferation of adventitial fibroblasts via α_2A_-AR [23]. Thus, we hypothesized that DEX may affect LPS-induced differentiation of CFs through activation of α_2_-AR, thereby accelerating cardiac fibrosis and potentially causing increased mortality in septic patients with DEX treatment.

Currently, there is no information on the role of DEX in CFs. Thus, the purpose of this study was to characterize the effects of DEX on the differentiation of CFs into myofibroblasts induced by LPS and the mechanism underlying these effects.

## 2. Results

### 2.1. DEX Promoted LPS-Induced Differentiation of CFs to Myofibroblasts In Vitro

**Figure 1:**
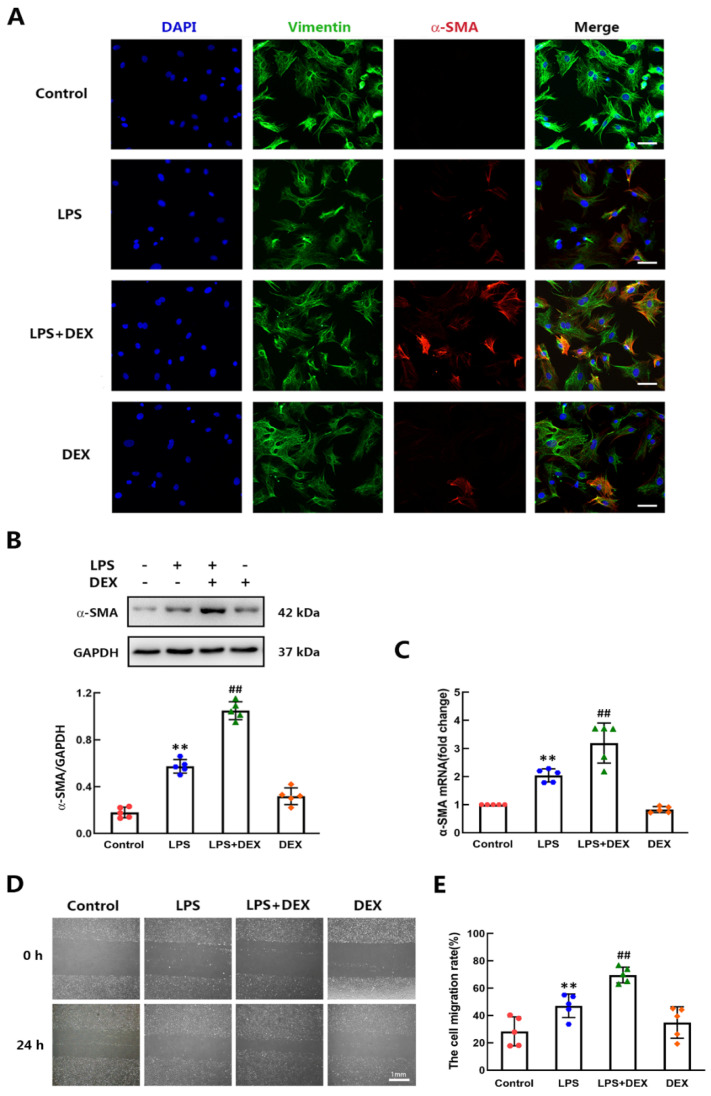
DEX promoted LPS-induced differentiation of CFs from wild-type adult mice. CFs were treated with vehicle and LPS at a concentration of 1 μg/mL for 15 min, followed by normal saline or DEX (0.1 μM) treatment. (**A**) Representative immunofluorescence imaging of CFs after LPS and/or DEX stimulation for 24 h. α-SMA (red)-labeled myofibroblasts, vimentin (green)-labeled CFs and nuclei were stained with DAPI (blue). Scale bar = 100 μm. (**B**) The protein level of α-SMA in CFs was measured by western blotting. (**C**) The mRNA level of α-SMA was assayed in CFs treated with LPS and/or DEX for 12 h. (**D**) The migration ability of CFs was assessed using scratch assay. The photographs were taken 24 h after the scratch of the confluently cultured CFs. (**E**) Quantification of the migration capacity of CFs. The migration rate was calculated by ImageJ software as described in the methods. Mean ± standard error of the mean (*n* = 5). ** *p* < 0.01 versus the control group. ^##^ *p* < 0.01 versus LPS group.

LPS is a potent inducer of fibroblast-to-myofibroblast differentiation, which is characterized by the high expression of α-SMA [6]. To determine the effect of DEX on LPS-induced differentiation of CFs to myofibroblasts, the protein expression of α-SMA in the LPS-administered CFs with or without DEX treatment was first detected by immunofluorescence staining and western blot. Previous reports have shown that CFs spontaneously differentiate into myofibroblasts under standard culture conditions [24]. Therefore, we performed our studies with no-passage fibroblasts. The fibroblastic phenotype was retained without LPS stimulation, as indicated by the low expression of α-SMA. As expected, the CFs treated with LPS for 24 h exhibited an increase in α-SMA expression compared with the control group. We observed a drastic upregulation of α-SMA expression in the DEX plus LPS-treated CFs compared to the LPS alone group. DEX slightly increased the expression of α-SMA in CFs without LPS stimulation, but the increase was not statistically significant (Figure 1A,B). We also measured the mRNA level of α-SMA in CFs after DEX stimulation for 12 h by using qPCR. The results showed that the mRNA level of α-SMA in CFs was much higher in the DEX plus LPS group than in the LPS group (Figure 1C). Activated fibroblasts migrate to the injured area, and the migration capacity of the fibroblasts can be assessed by the conventional scratch healing assay. In this study, the results showed that the treatment with LPS tended to reduce the cell-free scratch area. Furthermore, the LPS-mediated effect on migration capacity of CFs was significantly enhanced by DEX treatment (Figure 1D,E). These results indicated that DEX promotes LPS-induced differentiation of CFs to myofibroblasts in mice.

### 2.2. DEX Enhanced LPS-Induced Collagen I/III Synthesis and Phosphorylation of PKC, p38 and Smad2/3 in Mouse CFs

**Figure 2:**
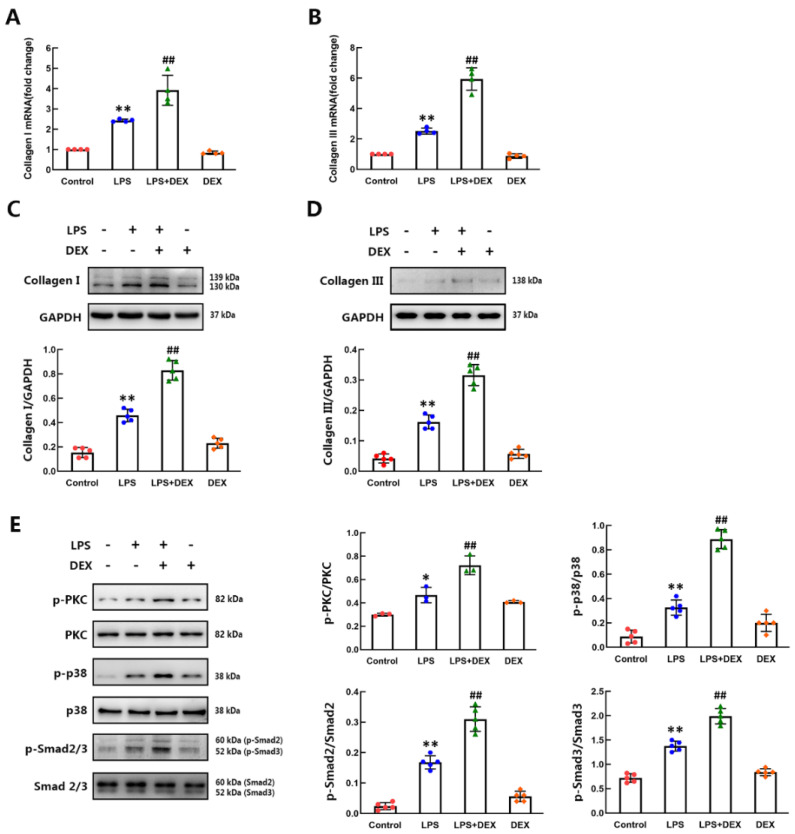
DEX accelerated collagen I/III synthesis and phosphorylation of PKC, p38 and Smad2/3 in CFs after LPS stimulation. (**A**,**B**) The mRNA levels of collagen I and collagen III in CFs after LPS and/or DEX stimulation for 12 h were detected by qPCR. (**C**,**D**) The protein levels of collagen I and collagen III in CFs treated with LPS and/or DEX for 24 h were measured by western blot. (**E**) The phosphorylation of PKC, p38 and Smad2/3 in CFs after LPS and/or DEX administration was detected by Western blot. Quantification of the relative protein levels in the panels next to the images. Mean ± standard error of the mean (*n* = 3–5). * *p* < 0.05, ** *p* < 0.01 versus control group. ^##^ *p* < 0.01 versus LPS group.

Following acute myocardial injury, myofibroblasts secrete elevated levels of collagen I/III [25]. To determine the effect of DEX on the synthesis of collagen I/III in CFs treated with LPS, we detected the mRNA and protein levels of collagen I and collagen III in CFs after DEX and LPS stimulation for 12 and 24 h separately by qPCR and Western blotting. Both the mRNA and protein expression levels of collagen I and collagen III in LPS-treated cells were significantly increased compared with those in control cells. DEX accelerated the LPS-induced increase in collagen I and collagen III in CFs (Figure 2A–D). Some studies have shown that the activation of the p38 and Smad2/3 cascades is essential for the development of cardiac fibrosis [26]. The accelerated effect of an α_2_-AR agonist on p38 phosphorylation was mediated by activation of PKC [27]. To investigate the downstream signaling pathways initiated by DEX and LPS, the levels of activated PKC, p38 and Smad2/3 were measured at 4 h after LPS administration. As determined by Western blotting, phosphorylated PKC (p-PKC), phosphorylated p38 (p-p38) and phosphorylated Smad2/3 (p-Smad2/3) levels were markedly upregulated following stimulation with LPS. DEX promoted LPS-induced phosphorylation of PKC, p38 and Smad2/3. Total PKC, p38 and Smad2/3 protein expression levels were also examined and found to be unaffected by DEX and LPS administration (Figure 2E). These results indicate that treatment with DEX after LPS administration enhances collagen I/III synthesis and activation of the PKC-p38-Smad2/3 signaling pathway in CFs.

### 2.3. The Role of DEX in LPS-Induced Differentiation of CFs Was Dependent on α_2A_-AR

**Figure 3:**
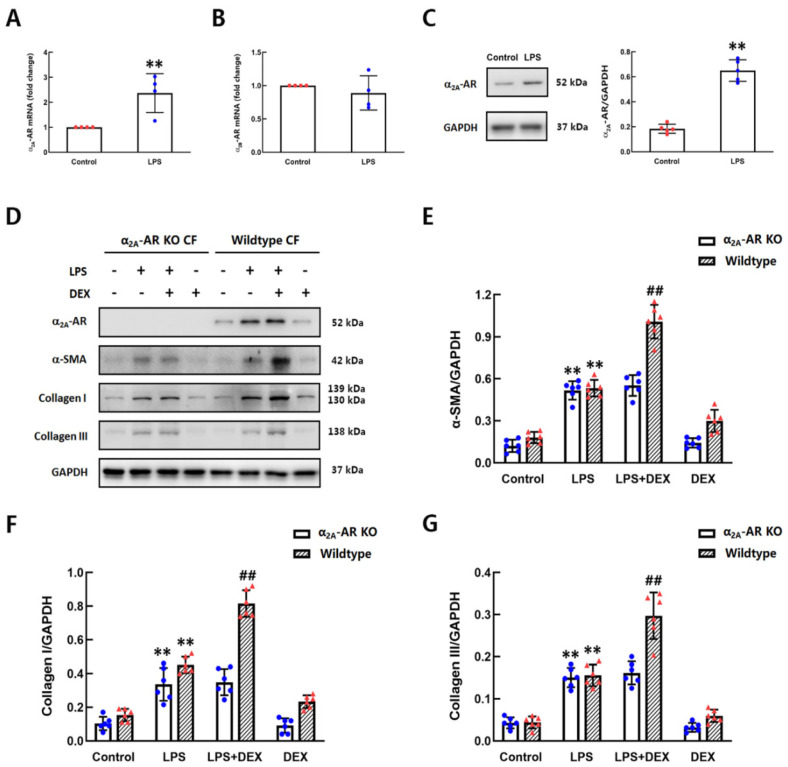
The effect of DEX on enhancing LPS-induced differentiation of CFs was dependent on α_2A_-AR. (**A**,**B**) The mRNA levels of α_2A_-AR and α_2B_-AR in wild-type CFs with and without LPS stimulation for 2 h. (**C**) The expression of α_2A_-AR in wild-type CFs after LPS treatment for 6 h. (**D**–**G**) The expressions of α-SMA and collagen I/III in CFs isolated from wild-type and α_2A_-AR knockout (KO) mice were detected by western blot. Quantification of the relative protein levels in the panels next to the images. Mean ± standard error of the mean (*n* = 4–6). ** *p* < 0.01 versus control group. ^##^ *p* < 0.01 versus LPS group.

The α_2_-ARs have been divided into three subtypes: α_2A_-AR, α_2B_-AR and α_2C_-AR. Previous studies have reported that only α_2A_-AR mRNA, but not α_B_-AR or α_2C_-AR, was detected in cultured adventitial fibroblasts of rat aortae [23]. To confirm the expression of α_2_-AR subtypes in CFs of mice, the mRNA levels of α_2A_-AR, α_2B_-AR and α_2C_-AR in CFs with and without LPS stimulation for 2 h were measured by qPCR. The mRNA expression of both α_2A_-AR and α_2B_-AR was detected, but α_2C_-AR was undetectable. Compared with control CFs, the mRNA level of α_2A_-AR was increased in LPS-treated CFs (Figure 3A,B). We also detected the protein level of α_2A_-AR in CFs after LPS administration for 6 h. As depicted in Figure 3C, the protein expression of α_2A_-AR was markedly higher in the LPS group than in the control group. To further investigate whether the facilitating effect of DEX on the differentiation of CFs is dependent on α_2A_-AR, CFs were isolated from wild-type and α_2A_-AR knockout (KO) mice, then treated with DEX and/or LPS. The CFs of α_2A_-AR KO mice treated with LPS showed significantly increased expression of α-SMA and collagen I/III, which are major markers of myofibroblasts. However, unlike the CFs of wild-type mice, DEX had no effect on the LPS-induced upregulation of α-SMA and collagen I/III in the CFs of α_2A_-AR KO mice (Figure 3D–G). It means that the effect of DEX on promoting LPS-induced differentiation of CFs into myofibroblasts is α_2A_-AR-dependent.

### 2.4. The Enhancing Effect of DEX on CF Differentiation Was TNF-α and IL-6 Independent

**Figure 4:**
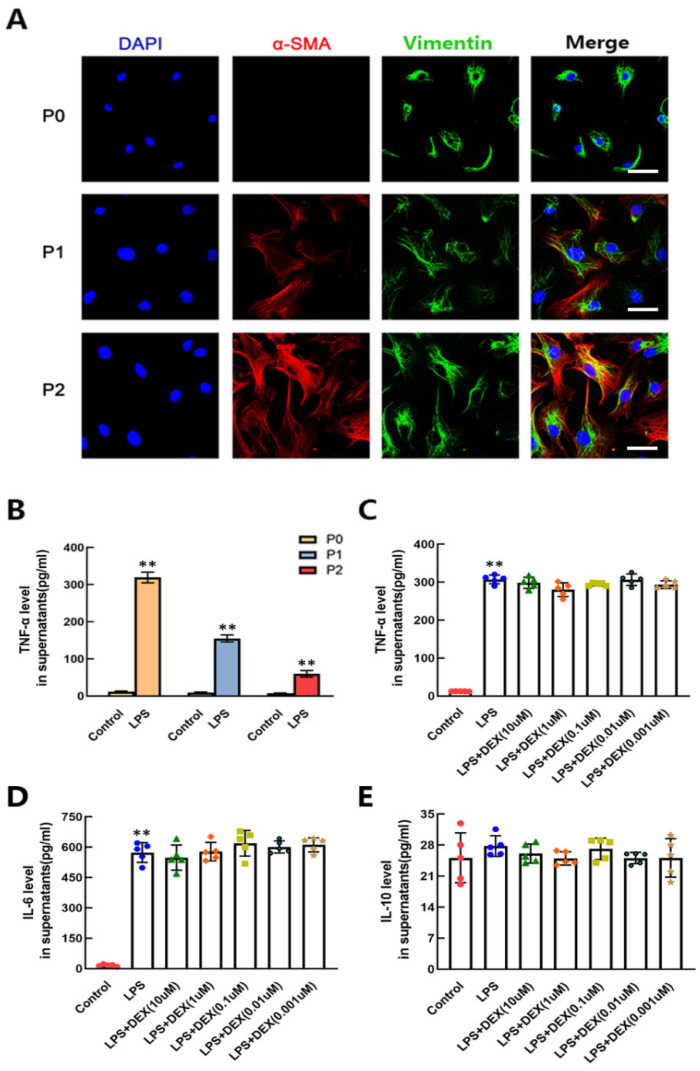
The accelerating effect of DEX on LPS-induced CF differentiation was TNF-α and IL-6 independent. (**A**) Immunofluorescent staining of primary CFs from nonpassage (P0) to second passage (P2). Scale bar = 100 μm. (**B**) The contents of TNF-α in CFs at different passages were determined by ELISA 24 h after LPS challenge. (**C**–**E**) The influence of DEX on TNF-α, IL-6 and IL-10 release from primary CFs treated with LPS. Mean ± standard error of the mean (*n* = 5). ** *p* < 0.01 versus the control group.

It has been reported that the differentiation of CFs occurs spontaneously with cell passage [24]. Therefore, we detected the expression of α-SMA in CFs from three different passages, from nonpassage to second passage, by immunofluorescence assay. The results showed that the expression of α-SMA increased as the number of CFs increased. The strongest expression of α-SMA was observed in the second passage of CFs (Figure 4A). To clarify the influence of LPS on CFs of different passages, we tested the TNF-α level of the cell supernatant after LPS stimulation for 24 h by ELISA. As shown in Figure 4B, the TNF-α release of nonpassage CFs after LPS stimulation was much higher than that in the first- and second-passage CFs, which had a weaker response to LPS. Other studies have shown that CFs play critical roles as intermediate sensors and amplifiers of inflammatory signals in response to LPS through the production of cytokines, such as TNF-α [28]. Meanwhile, the injurious elements that induce the release of TNF-α and IL-6 from immune cells and myocytes may contribute to the differentiation of CFs to myofibroblasts [29]. To investigate whether the effect of DEX on promoting the differentiation of CFs is associated with the release of cytokines, we detected the levels of TNF-α, IL-6 and IL-10 in nonpassage CFs after LPS and different concentrations of DEX stimulation by ELISA. The results showed that there was no effect of different concentrations of DEX on LPS-induced TNF-α and IL-6 release (Figure 4C,D). The levels of IL-10 did not change significantly in CFs treated by LPS with or without DEX stimulation (Figure 4E). These data indicate that the effect of DEX on promoting the differentiation of CFs was not dependent on TNF-α and IL-6. The sensitivity of CFs to endotoxin was decreased with the deepening of differentiation, resulting in rare production of TNF-α after LPS stimulation.

### 2.5. DEX Accelerated LPS-Induced Differentiation of CFs to Cardiac Myofibroblasts by Activating the PKC-p38-Smad2/3-Mediated Signaling Pathway

**Figure 5:**
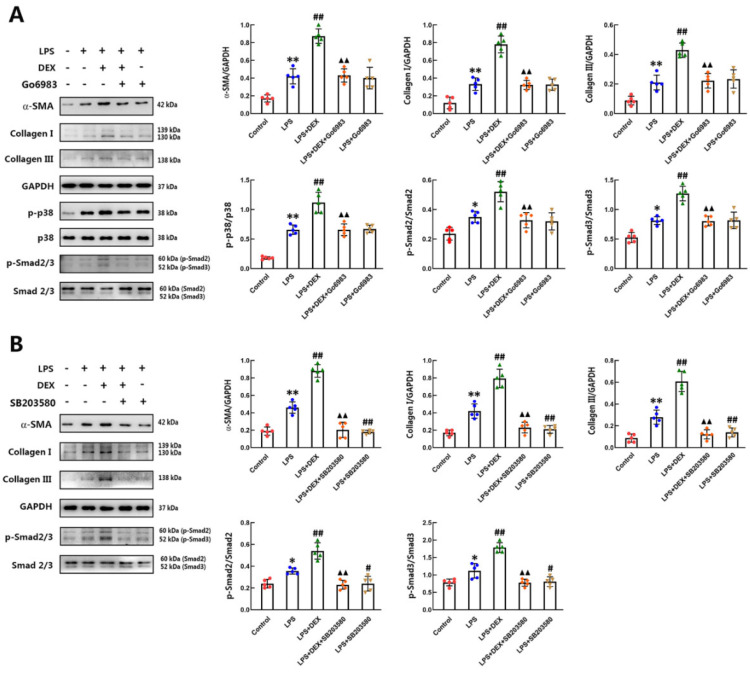
The effects of PKC and p38 inhibitor on DEX-promoted differentiation of CFs after LPS stimulation. (**A**) The PKC inhibitor Go6983 was used to treat CFs 30 min prior to DEX and LPS stimulation. The expression of α-SMA and collagen I/III, as well as the phosphorylation of p38 and Smad2/3, were detected by Western blot. (**B**) CFs were preincubated with SB203580, a selective inhibitor of p38, for 30 min before treatment with LPS and DEX. The levels of α-SMA, collagen I/III and phosphorylation of Smad2/3 were measured by Western blot analysis with antibodies binding to the proteins. Quantitative analyses of the relative protein levels are plotted in the panels on the right of image. Mean ± standard error of the mean (*n* = 5). * *p* < 0.05, ** *p* < 0.01 versus control group. ^#^ *p* < 0.05, ^##^ *p* < 0.01 versus LPS group. ^▲▲^ *p* < 0.01 versus LPS + DEX group.

We have demonstrated that the phosphorylation of PKC, p38 and Smad2/3 was involved in DEX- and LPS-induced CF differentiation. To further confirm the role of PKC in the mechanisms for enhancing the effect of DEX on LPS-induced differentiation of CFs, we used an inhibitor of PKC, Go6983, to treat CFs 30 min prior to DEX and LPS stimulation. The expression of α-SMA and collagen I/III, as well as the phosphorylation of p38 and Smad2/3 were detected by western blotting. As depicted in Figure 5A, inhibition of PKC activation with 1 μM Go6983 reversed the enhancing effects of DEX on α-SMA and collagen I/III expression, as well as p38 and Smad2/3 activation in LPS-challenged CFs, while pretreatment with Go6983 did not affect the expression of α-SMA and collagen I/III in CFs treated with LPS alone. Furthermore, we also confirmed the role of p38 downstream of the PKC-mediated signaling pathway by using SB203580, a selective inhibitor of p38. CFs were preincubated with SB203580 for 30 min before treatment with LPS and DEX. The results showed that the effect of DEX on LPS-treated CFs was completely blocked by SB203580 pretreatment. Moreover, SB203580 markedly prevented LPS-induced high expression of α-SMA and collagen I/III and phosphorylation of Smad2/3 in CFs (Figure 5B). These data indicate that the effect of DEX on promoting LPS-induced differentiation of CFs was mediated by the PKC-p38-Smad2/3 signaling pathway.

### 2.6. DEX Aggravated LPS-Induced Cardiac Fibrosis in Mice

**Figure 6:**
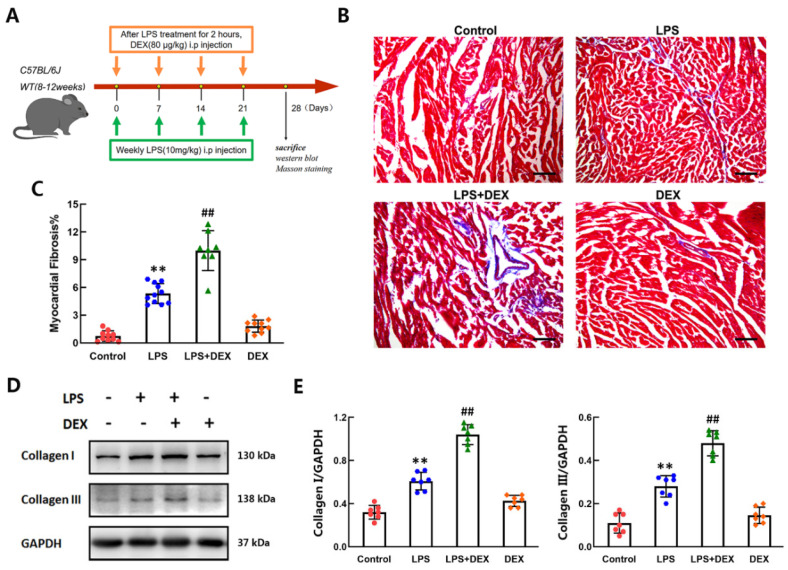
DEX aggravated cardiac fibrosis in LPS-challenged mice. (**A**) Mice were injected i.p. with LPS at a dose of 10 mg/kg once a week for four weeks. The control group received 0.3 mL of saline instead of LPS. DEX (80 μg/kg) was given i.p. after LPS treatment for 2 h every week. After four weeks, the mice were anaesthetized with diethyl ether and sacrificed. Then, the left ventricles of hearts were obtained for western blot and Masson’s staining analysis. (**B**) Representative images of myocardial tissue sections stained with Masson trichrome showing interstitial and perivascular collagen deposition. Scale bar = 200 μm. (**C**) Quantification of the fractional area of cardiac fibrosis based on five randomly selected fields in the stained myocardial tissue sections. (**D**) The levels of collagen I and collagen III in the myocardium of LPS-challenged mice were detected by Western blot. (**E**) Quantitative analyses of the relative protein levels were plotted. Mean ± standard error of the mean (*n* = 7–11). ** *p* < 0.01 versus control group. ^##^ *p* < 0.01 versus LPS group.

Previous reports have shown that recurrent exposure to subclinical LPS induces cardiac fibrosis and increases mortality, which occurs commonly in a murine model [30,31]. To assess whether DEX enhances LPS-induced cardiac fibrosis in vivo, mice were injected i.p. with LPS at a dose of 10 mg/kg once a week for four weeks, and DEX (80 μg/kg) was given i.p. after LPS treatment for 2 h every week. After four weeks, fibrosis and collagen I/III production in the hearts of mice were detected by Masson’s trichrome staining and western blotting analysis (Figure 6A). The results of Masson staining in the four groups are shown in Figure 6B,C. After Masson staining, cardiomyocytes were stained red, whereas collagen fibers were stained blue. More collagen deposition was observed in the hearts of mice administered LPS compared with the controls. Ratios of collagen area to total area were measured. The fractional area of cardiac fibrosis was larger in the LPS group than in the controls, and the difference between groups was significant. In comparison with the LPS group, fibrosis of the heart was significantly enhanced by co-stimulation with DEX and LPS. The Western blotting results revealed that the levels of collagen I and collagen III in CFs in LPS-treated mice were significantly increased compared with those in control mice. The effect of LPS on collagen I/III expression was enhanced by DEX treatment (Figure 6D,E). However, there were no significant differences in cardiac fibrosis or collagen I/III levels between the control and DEX groups. These results demonstrated that DEX aggravated LPS-induced cardiac fibrosis and collagen I/III production in mice.

## 3. Discussion

DEX is an α_2_-AR agonist that presents sympatholytic action in certain parts of the brain with anxiolytic, sedative, and pain-relieving effects [8]. It was first approved in December 1999 by the FDA for use in the ICU and procedural sedation in adults. Severe sepsis is a major cause of death worldwide and the most common cause of death among critically ill patients [32]. The prevalence of sepsis is estimated at 18 million cases per year worldwide [33], with three cases in every 1000 patients of the population in the USA [34]. Many studies suggest that DEX reduces mortality and cardiac injury in septic experimental animals. However, clinical trials have shown that DEX, when used for sedation in mechanically ventilated adults, may increase 90-day mortality in septic patients [10]. Previous data from our laboratory have also shown that α_2_-AR antagonists attenuate mortality and cardiac dysfunction in septic mice, and α_2A_-AR blockade significantly reduces cardiopathy in septic rats [35,36]. It has been confirmed that α_2_-AR is expressed on fibroblasts, an important component of heart tissue [23]. Therefore, we speculated that DEX might enhance LPS-induced cardiotoxic effects by acting on cardiac fibroblasts. The results of the present research, which found that DEX markedly promoted the progression of LPS-activated CF differentiation and cardiac fibrosis in mice, proved our hypothesis.

Fibroblasts are a general term that encapsulates cells in connective tissue that synthesize collagen and other ECM components [37]. The term myofibroblast has been used to differentiate a fibroblast cell type that is α-SMA positive [3]. Increasing evidence has demonstrated that many of the functional effects of CF are mediated through the differentiation of CF to myofibroblasts, which produce more ECM proteins, such as collagen I and collagen III, leading to cardiac fibrosis and impairing cardiac function [38,39,40,41]. To confirm the effect of DEX on LPS-stimulated CFs, we decided to focus on the differentiation of CFs at 24 h after LPS and DEX treatment. It demonstrated that DEX posttreatment promoted the LPS-induced differentiation of CFs to myofibroblasts, characterized by an abundance of α-SMA and collagen I/III expression at both the mRNA and protein levels. Furthermore, our results indicated the involvement of PKCp38-Smad2/3 signaling pathways in the facilitating effect of DEX on CF differentiation after LPS stimulation. Three α_2_-AR subtypes, α_2A_, α_2B_ and α_2C__,_ have been identified on the basis of pharmacological properties and molecular cloning evidence [42]. The subtypes of α_2_-AR are involved in various physiological functions dependent on distribution and density, particularly in the cardiovascular system as well as in the central nervous system [43]. Although α_2_-AR is not found in cardiomyocytes, α_2A_-AR mRNA is present in α_2A_-AR in adventitial fibroblasts of rat aortae [23]. We found that both α_2A_-AR and α_2B_-AR were well expressed in cultured CFs but not α_2C_-AR. The mRNA expression of α_2C_-AR was not detected at all. Interestingly, our results indicated that LPS had no significant effects on the mRNA level of α_2B_-AR but markedly promoted the mRNA and protein expression of α_2A_-AR in CFs. In addition, the differentiation of CFs was not induced by DEX alone. These data suggested that DEX is likely to promote LPS-induced differentiation of CFs through specific activation of α_2A_-AR receptors, which is increased after LPS challenge. CFs isolated from α_2A_-AR KO mice were used to prove this speculation. The current study showed that LPS can also upregulate the production of α-SMA and collagen I/III in CFs without α_2A_-AR expression. However, the promotional effect of DEX on LPS-induced differentiation disappeared in the CFs of α_2A_-AR KO mice. Our results indicated that the role of DEX in LPS-stimulated CF differentiation is dependent on α_2A_-AR.

LPS mediates p38 activation by binding to Toll-like receptors [44]. However, little is known about the coupling of α_2_-AR to p38-Samd2/3 signaling pathways in CFs. Previous research indicated that α_2_-AR agonists such as clonidine significantly enhanced interleukin-12 production in mouse macrophages via a PKC-p38 signaling pathway [27]. In this report, the phosphorylations of PKC, p38 and Smad2/3 were found to be required in LPS-stimulated differentiation of CFs with or without DEX treatment. We further confirmed the sequentially activated PKC and p38 participate in the promoting effects of DEX on LPS-induced differentiation of CFs by using inhibitors. Our results showed that the PKC inhibitor Go6983 suppressed DEX-enhanced production of α-SMA and collagen I/III, as well as p38 and Smad2/3 phosphorylation, in CFs treated with LPS and DEX. However, Go6983 did not affect the differentiation of CFs after LPS challenge alone. Furthermore, inhibition of p38 completely eliminated α-SMA and collagen I/III synthesis, as well as the phosphorylation of Smad2/3, in LPS-treated CFs and CFs treated with or without DEX. Our findings provide the first evidence that DEX-mediated activation of PKC promotes LPS-induced p38 and Smad2/3 phosphorylation, leading to increased α-SMA and collagen I/III production.

It was reported that LPS could induce TNF-α gene transcription and the production of biologically active TNF-α in neonatal rat cardiac fibroblasts [45,46]. Wang et al. demonstrated that norepinephrine promotes LPS-induced TNF-α production in Kupffer cells of the liver by stimulating α_2A_-AR [47]. In the present study, the results showed that CFs at different passages responded differently to LPS. After LPS stimulation, the TNF-α released from the nonpassage CFs was much higher than that in the first and second passages of CFs that had little response to LPS. This result indicated that the response of CFs to LPS stimulation was weakened with differentiation to myofibroblasts. We also observed CF differentiation to myofibroblasts with increased cell passage under normal culture conditions, which was in accordance with a previous report [24]. To our surprise, DEX at different concentrations did not affect LPS-induced TNF-α and IL-6 productions from CFs. This may be related to DEX promoting LPS-induced differentiation of CFs, which had low sensitivity to endotoxin stimulation. More studies are still needed to understand the mechanisms of low responses of myofibroblasts to LPS in the future.

In addition, we examined the effect of DEX on cardiac fibrosis in a mouse model of chronic LPS stimulation. Our present results demonstrated that DEX enhanced LPS-induced cardiac fibrosis and collagen I/III synthesis in vivo. Because of the complexity of the situation in vivo, in addition to activating α_2A_-AR-mediated PKC-p38-Smad2/3 signaling pathway, the facilitating effect of DEX on LPS-induced cardiac fibrosis in animal model could be due to other possible mechanisms. The identical may not work exactly the same in different animal models. People have reported cardioprotective effects of DEX on myocardial infarction such as reduction of fibrosis and inflammation [48]. It is still being studied how DEX, a highly selective α_2_-AR agonist, plays an anti-fibrosis role in myocardial infarction. Furthermore, no research has shown that the expression of α_2A_-AR receptor in the heart is increased during myocardial infarction. Endotoxin-induced cardiac injury is significantly different from myocardial infarction. In our research, LPS induces increased expression of α_2A_-AR on cardiac fibroblast, which provides an important basis for DEX to come into play. DEX may promotes LPS-induced myocardial fibroblast differentiation through activation of increased α_2A_-AR receptor in vivo. More studies are needed to dissect further the role of DEX in animal model for future clinical trials.

## 4. Materials and Methods

### 4.1. Materials

LPS (Escherichia coli, 055: B5) and DEX (SML0956) were purchased from Sigma Aldrich (SML0956, St. Louis, MO, USA). Go6983 (HY-13689) and SB203580 (HY-10256) were obtained from MedChemExpress (Monmouth Junction, NJ, USA). Anti-α-SMA (ab7817), p-PKC (ab63387) and anti-α_2A_-AR (ab45871) antibodies were obtained from Abcam (Cambridge, UK). Antibodies against vimentin (#5741), p38 (#9212S), p-p38 (#4511S), PKC (2683S) and GAPDH (#2118) were purchased from Cell Signaling Technology, Inc. (Beverly, MA, USA). Antibodies recognizing Smad 2/3 (sc-133098) were purchased from Santa Cruz Biotechnology, Inc. (Santa Cruz, CA, USA). Antibodies against collagen I (WL0088), collagen III (WL03186), and p-Smad 2/3 (WL02305) were obtained from Wanleibio (Shenyang, China). Alexa Fluor 647-labeled goat antibody against mouse IgG (A21235), Alexa Fluor 488-labeled goat antibody against rabbit IgG (A21206), goat anti-rabbit IgG antibody (#31462) and goat anti-mouse IgG antibody (#31438) were obtained from Thermo Fisher Scientific (Logan, UT, USA). The enzyme-linked immunosorbent assay (ELISA) kits for the detection of mouse TNF-α (MTA00B), interleukin (IL)-6 (M6000B) and IL-10 (M1000B) were purchased from R&D Systems (MTA00B, R&D Systems, Minneapolis, MN, USA).

### 4.2. Adult Mouse Cardiac Fibroblast Culture and Treatment

Cardiac ventricles were excised from 6- to 8-week-old male C57Bl/6J mice, thoroughly minced with sterile fine scissors and digested in 10 mL HBSS containing 0.1% collagenase type 2 (C6885-5G, Sigma Aldrich) at 37 °C. During this incubation, the digesting tissue was triturated for one min with a sterile serological pipette every 5 min. The supernatant cell suspension containing liberated fibroblasts was then collected into a tube containing cold Dulbecco’s modified Eagle’s medium (DMEM). The undigested fraction was reconstituted with fresh digestion media, and the same digestion procedure was repeated till almost the entire tissue was dissociated into single cells. The cell fraction was collected after a final centrifugation at 500× *g* for 10 min at 4 °C, resuspended in DMEM supplemented with 10% FBS and plated in a 6-well plate (1.0 × 10^5^ cells/well). After 48 h of culture, CFs were treated with vehicle and LPS at a concentration of 1 μg/mL for 15 min, followed by normal saline or DEX (0.1 μM) treatment. In separate experiments, CFs were preincubated with Go6983 (1 μM, a selective PKC inhibitor) or SB203580 (1 μM, a selective inhibitor of p38) for 30 min before treatment with LPS. Moreover, cell viability was measured using Cell Counting Kit-8 (Dojindo Molecular Technologies Inc., Kumamoto, Japan).

### 4.3. Animals and Treatment Protocols

Male C57Bl/6J mice (8–10 weeks old) weighing 25 to 28 g were obtained from Guangdong Experimental Animal Center. α_2A_-AR knockout (α_2A_-AR KO, 25–28 g) mice were generated by Adra2a^tm1Bkk^ mice (Strain Name: B6.129-Adra2a^tm1Bkk^/J), which were obtained from Jackson Laboratory (Bar Harbor, ME, USA). All animals were given free access to standard mouse chow and water and maintained at room temperature (24-C T 2-C) with a 12-h light-dark cycle. The mice were then randomly assigned to one of the following four groups: control, LPS, LPS + DEX and DEX. Mice were injected i.p. with LPS at a dose of 10 mg/kg once a week for four weeks. The control group received 0.3 mL of saline instead of LPS. DEX (80 μg/kg) was given i.p. after LPS treatment for 2 h every week. After 4 weeks, the mice were anaesthetized with diethyl ether and sacrificed. Then, the left ventricles of hearts were obtained for western blot and Masson’s staining analysis. All animal experiments were accomplished according to the guidelines for the Care and Use of Laboratory Animals of the National Institutes of Health. Our study was approved by the Ethics Committee of Jinan University.

### 4.4. Immunofluorescence Staining

Adult mouse cardiac fibroblasts with/without LPS or DEX stimulation were fixed with 4% formalin solution and then permeabilized with PBS containing 0.1% Triton X-100. After three washes with PBS, the cells were blocked in PBS with 1% BSA and 0.1% Triton X-100, followed by incubation with a 1:200 dilution of antibodies against α-SMA and vimentin at 4 °C overnight. The cells were then incubated with the secondary anti-rabbit Alexa Fluor 488 antibody for vimentin and secondary anti-mouse Alexa Fluor 647 antibody for α-SMA at room temperature for 1 h. The cells were then washed three times with PBS and incubated with a 1:200 dilution of 4′,6-diamidino-2-phenylindole (DAPI) for 15 min. The cells were observed with a Leica fluorescence microscope.

### 4.5. Western Blotting Analysis

Western blot experiments were performed as previously described [49]. Cells and heart tissues were lysed in RIPA buffer. Proteins were separated on SDS-polyacrylamide gels by electrophoresis and then transferred to PVDF membranes. The membrane was blocked with TBST buffer (20 mM Tris–HCl, 137 mM NaCl, and 0.1% Tween 20, pH 7.5) with 5% skim milk at room temperature for 1 h. Then, the membranes were incubated with primary and secondary antibodies. Afterward, the immunoblotted proteins were visualized with enhanced chemiluminescence (ECL) reagents. ImageJ software, an open-source image processing program, was used to quantify blots.

### 4.6. Quantitative Real-Time Polymerase Chain Reaction (qPCR) Analysis

qPCR was used to measure the mRNA levels of α_2A_-AR, α_2B_-AR and α_2C_-AR in mouse CFs. Total RNA was isolated from cells using TRIzol reagent according to the manufacturer’s instructions. RNA samples were reverse-transcribed using random hexamer primers in the presence of RNase inhibitor (Takara Bio, Shiga, Japan). cDNA was amplified using ChemoHS qPCR mix (Monad), 0.2 μM of each primer and nuclease-free water. Amplified cDNA signals were detected and analyzed by CFX Maestro Software v1.1 (Bio–Rad, Hercules, CA, USA) using β-Actin as an endogenous control. The results are expressed as the fold increase over the control. The specific primers used in this study were as follows. α_2A_-AR: forward 5′-GTGACACTGACGCTGGTTTG-3′, reverse 5′-CCAGTAACCCATAACCTCGTTG-3′. α_2B_-AR: forward 5′-TCTTCACCATTTTCGGCAATGC-3′, reverse 5′-AGAGTAGCCACTAGGATGTCG-3′. α_2C_-AR: forward 5′-CTGTGGTGGGTTTCCTCATCG-3′, reverse 5′-ACTTGCCCGAAGTACCAGTAG-3′. α-SMA: forward 5′-TTCCTTCGTGACTACTGCCG-3′, reverse 5′-TATAGGTGGTTTCGTGGATGCC-3′. Collagen I: forward 5′-CCCTGAAGTCAGCTGCATACACAA-3′, reverse 5′-CCTACATCTTCTGAGTTTGGTGAT-3′. Collagen III: forward 5′-GAGATGTCTGGAAGCCAGAACCAT-3′, reverse 5′-GATCTCCCTTGGGGCCTTGAGGT-3′. β-actin: forward 5′-GGCTGTATTCCCCTCCATCG-3′, reverse 5′-CCAGTTGGTAACAATGCCATGT-3′.

### 4.7. Assays for Concentrations of TNF-α, IL-6 and IL-10

Adult mouse cardiac fibroblasts were treated with LPS for 24 h, and then, the culture supernatants were collected. The concentrations of TNF-α, IL-6 and IL-10 in the supernatant were measured by using a commercially available ELISA kit according to the manufacturer’s instructions.

### 4.8. Masson’s Trichrome Stain Analysis

For evaluation of cardiac fibrosis, the left ventricles were embedded in 10% formalin and sliced at 5 μm. The tissue sections were stained with Masson’s trichrome according to the manufacturer’s instructions and then examined under light microscopy. After Masson staining, cardiomyocytes were stained red, whereas blue staining indicated positive staining for collagen. The fibrotic changes were assessed by a pathologist in 10 randomly selected high-power fields for each tissue slide. ImageJ software was used to quantify the fractional area of cardiac fibrosis (collagen area/total area).

### 4.9. Migration Assay

An in vitro scratch assay was used to assess the migration capacity of cells. CFs were seeded into 6-well plates at a density of 5 × 10^5^ cells/well. After 24 h of incubation, the cell monolayer was scratched with a sterile pipette tip. Then, the cells were incubated with vehicle and LPS for 15 min, followed by normal saline or DEX (0.1 μM) treatment for 24 h. The scratch areas were photographed using an IX51 Inverted Phase Contrast Fluorescence Microscope (Olympus, Tokyo, Japan) and quantified by ImageJ software. The relative migration ratio of the cells was calculated according to the formula: (size at 0 h–size at 24 h)/size at 0 h.

### 4.10. Statistical Analysis

Mean densitometry values and all other quantitative data are presented as the mean ± standard error of the mean (SEM). Comparisons among groups were made using Student’s *t*-test or one-way ANOVA followed by the Student-Newman–Keuls test. Differences with *p* values < 0.05 were considered statistically significant. SPSS software was used to analyze the data.

## 5. Conclusions

**Figure 7:**
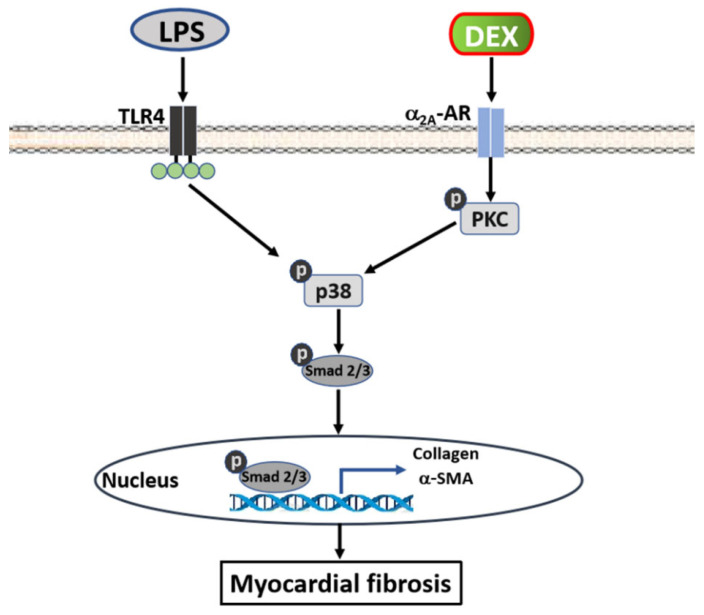
Proposed signaling mechanisms of DEX promote LPS-induced differentiation of CFs and cardiac fibrosis. DEX initiates α_2A_-AR-mediated activation of PKC, which accelerates LPS-induced phosphorylation of p38 and Smad2/3 in CFs. This subsequently upregulates α-SMA and collagen I/III expression, resulting in cardiac fibrosis.

In summary, this is the first study to demonstrate that LPS increased α_2A_-AR expression in CFs. Accordingly, the intensified effect of DEX on LPS-induced differentiation of CFs is mediated by accelerating the activation of the PKC-p38-Smad2/3 signaling pathway through increasing α_2A_-AR (Figure 7). Our study provides evidence that the clinical application of DEX may cause unfavorable side effects in septic patients by enhancing cardiac fibrosis. This may provide new insight into the choice of sedatives for septic patients in the ICU.

## Data Availability

The data that support the findings of this study are openly available on request from the corresponding author.
